# The Prevalence of Multidrug Resistance of *Helicobacter pylori* and Its Impact on Eradication in Korea from 2017 to 2019: A Single-Center Study

**DOI:** 10.3390/antibiotics9100646

**Published:** 2020-09-27

**Authors:** Jae Yong Park, Tae-Seop Shin, Ji Hyun Kim, Hong Jip Yoon, Beom Jin Kim, Jae Gyu Kim

**Affiliations:** 1 Department of Internal Medicine, Chung-Ang University College of Medicine, Seoul 06974, Korea; jay0park@cau.ac.kr (J.Y.P.); redhouse@caumc.or.kr (H.J.Y.); kimbj@cau.ac.kr (B.J.K.); 2 MD Healthcare Inc., Seoul 03923, Korea; tsshin@postech.ac.kr; 3 Research Institute, Chung-Ang University, Seoul 06974, Korea; godkimjieun@cau.ac.kr

**Keywords:** *Helicobacter pylori*, antibiotic resistance, eradication, Korea

## Abstract

Antimicrobial resistance is one of the major factors determining the efficacy of *Helicobacter pylori* eradication therapy. This study aimed to estimate the recent prevalence of multidrug resistance of *H. pylori* and its impact on eradication in Korea. A total of 174 patients were prospectively enrolled at Chung-Ang University Hospital from 2017 to 2019. *H. pylori* strains were isolated from the gastric body and antrum. The minimum inhibitory concentrations of antibiotics were determined by the serial twofold agar dilution method. Eradication results were reviewed and analyzed in connection with antibiotic resistance. The prevalence of *H. pylori* infection was 51.7% (90/174). The culture success rate was 77.8% (70/90). The resistance rates for clarithromycin, metronidazole, amoxicillin, tetracycline, levofloxacin, and moxifloxacin were 28.6% (20/70), 27.1% (19/70), 20.0% (14/70), 18.6% (13/70), 42.9% (30/70), and 42.9% (30/70), respectively. The multidrug resistance (resistance to two or more classes of antimicrobials) rate was 42.9% (30/70). Dual resistance to clarithromycin and metronidazole was confirmed in 8.6% (6/70). Eradication with a first-line treatment was successful in 75% (36/48), and those who received second-line treatment all achieved successful eradication. The rate of multidrug resistance is increasing, and standard triple therapy (STT) is no longer an acceptable first-line option for *H. pylori* eradication in Korea.

## 1. Introduction

*Helicobacter pylori* is a well-known risk factor for gastric cancer as well as various other gastrointestinal diseases [1]. Eradication of *H. pylori* can prevent the recurrence of peptic ulcer disease, metachronous gastric cancer after resection of gastric cancer, and even induce the remission of gastric mucosa-associated lymphoid tissue lymphoma [2,3,4]. Recent guidelines have shown attempts to expand the indications for testing and treatment of *H. pylori* infection [5,6]. In line with these trends, the Korean government insurance system expanded its coverage for eradication therapy to include *H. pylori* gastritis since 2018. However, although regionally variable, *H. pylori* resistance rates to some key antibiotics are increasing in most parts of the world [7]. This antimicrobial resistance is becoming a major clinical problem undermining the efficacy of eradication regimens which had shown good effects in the past.

The efficacy of standard triple therapy (STT) with a proton pump inhibitor, amoxicillin, and clarithromycin is continuously decreasing in many countries worldwide [8]. Recent reports in Korea also have repeatedly shown that the efficacy of this popular regimen decreased to a suboptimal level [9,10]. As clarithromycin resistance is a decisive factor affecting the efficacy of STT, this regimen is not recommended in regions with high clarithromycin resistance [5,7]. In this scenario, resistance profile to metronidazole or fluoroquinolones is an important factor determining the efficacy of popular regimens other than STT, such as bismuth quadruple therapy (BQT) or fluoroquinolone-containing therapy [11]. When clarithromycin resistance is anticipated, physicians can consider alternative therapeutic regimens according to the metronidazole resistance at a community level. Fluoroquinolone-containing therapies should only be carefully selected considering that fluoroquinolone resistance greatly decreases the efficacy of the regimens including fluoroquinolones [12].

It is very important to have a good understanding of regional antibiotic resistance patterns of *H. pylori* for the clinical decision of effective empirical eradication therapies and adequate rescue therapies. Our study aimed to investigate the primary antibiotic resistance patterns of *H. pylori* and their impact on eradication in Seoul during 2017–2019, and to provide a basis for establishing an optimal strategy for the eradication therapy.

## 2. Results

### 2.1. Patient Demographics and the Culture Success Rate of H. pylori

A total of 174 patients were initially enrolled. The baseline characteristics of this population are shown in Table 1. The mean age of the subjects was 60.5 ± 13.1 years (range: 21–82), and 57.5% were male. The most common endoscopic diagnosis was gastritis (50.6%). Among 174 patients initially enrolled, 51.7% (90/174) showed positive test results for *H. pylori* infection. Among these patients with confirmed *H. pylori* infection, the culture success rate was 77.8% (70/90). Minimum inhibitory concentration (MIC) levels were evaluated in this group of 70 patients with successful *H. pylori* culture.

### 2.2. The Antibiotic Resistance Rates and MIC Profiles of H. pylori

**Figure 1:**
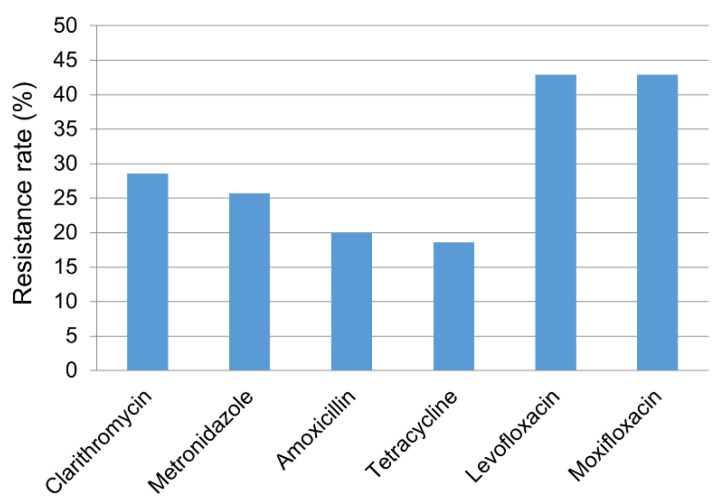
Antibiotic resistance profile of *Helicobacter pylori* strains (n = 70) for six antibiotic agents.

**Figure 2:**
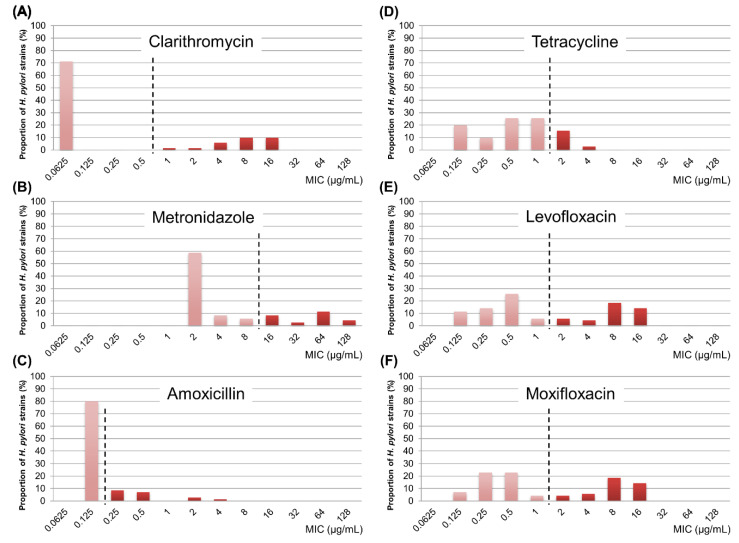
Distribution of minimum inhibitory concentration values for each antibiotic agent. (**A**) clarithromycin, (**B**) metronidazole, (**C**) amoxicillin, (**D**) tetracycline, (**E**) levofloxacin, (**F**) moxifloxacin. Vertical dashed lines indicate resistance breakpoints for each antibiotic agent. MIC, minimum inhibitory concentration.

The resistance rates for clarithromycin, metronidazole, amoxicillin, tetracycline, levofloxacin, and moxifloxacin were 28.6% (20/70), 27.1% (19/70), 20.0% (14/70), 18.6% (13/70), 42.9% (30/70), and 42.9% (30/70), respectively (Figure 1). In all, 70.0% (49/70) of patients showed resistance to one or more of the six antibiotic agents. We also investigated distribution of MIC values for each antibiotic agent (Figure 2). While the clarithromycin MIC was 0.0625 μg/mL in 71.4% of the isolated strains, most of the resistant strains showed MIC range of 4–16 μg/mL, showing a clear dual peak pattern. The MIC values for metronidazole were distributed over a wide range of 2–128 μg/mL. Strains with levofloxacin resistance always showed simultaneous resistance to moxifloxacin, suggesting cross-resistance among fluoroquinolones.

### 2.3. Differences of Antibiotic Resistance According to the Site of Isolation

Further analysis was performed to investigate how often antibiotic resistance patterns differ between the *H. pylori* strains isolated from different locations (antrum and body) of the stomach in the same patient. When one strain was susceptible to a certain antibiotic agent, while the other strain from the other site was resistant in the same host, discordant resistance was judged to be present. Among 52 patients with successful isolation of *H. pylori* strains from both the antrum and the body of the stomach, 26.9% (14/52) of the patients showed discordant antimicrobial resistant profiles between these anatomic areas. For each specific antibiotic agent, the discordant rates of *H. pylori* resistance to clarithromycin, metronidazole, amoxicillin, tetracycline, levofloxacin, and moxifloxacin according to different anatomic locations were 3.8% (2/52), 5.8% (3/52), 0% (0/52), 15.4% (8/52), 5.8% (3/52), and 5.8% (3/52), respectively (Table S1). Among the 52 patients, 4 (7.7%) patients had a strain susceptible to all the antibiotic agents in one site, while the strain from the other site showed resistance to one or more of the drugs. These four resistant strains comprised two strains resistant to tetracycline, one strain resistant to clarithromycin, one strain resistant to tetracycline, levofloxacin, and moxifloxacin.

### 2.4. Profile of Multidrug Resistance among H. pylori Strains

Subjects showing resistance to two or more classes of antimicrobial agents were regarded as having multidrug resistance. The multidrug resistance rate was 42.9% (30/70) in the culture success group, and 61.2% (30/49) in the patients with resistant *H. pylori* strains only, when considering all five classes of antimicrobial agents (amoxicillin, clarithromycin, metronidazole, tetracycline, and fluoroquinolone) (Table 2). As fluoroquinolone-containing therapy is generally recommended as a rescue therapy, not first-line therapy, we have also analyzed the multidrug resistance without fluoroquinolone (Table 3). The multidrug resistance rate was 22.9% (16/70) in the culture success group, and 36.4% (16/44) in the patients with resistant *H. pylori* strains only, when considering four of these major antibiotic classes except for fluoroquinolone. Detailed information of resistance profile is shown in Table S2. We have also analyzed the presence of simultaneous resistance to amoxicillin or metronidazole in addition to clarithromycin resistance, due to their importance in the efficacy of STT or BQT, which are very popular primary or secondary regimens. The dual resistance to clarithromycin and amoxicillin was present in 10.0% (7/70), and the dual resistance to clarithromycin and metronidazole was 8.6% (6/70). Clarithromycin-resistant *H. pylori* strains had 35% (7/20) and 30% (6/20) chance of carrying simultaneous resistance to amoxicillin and metronidazole, respectively.

### 2.5. The Effect of Antibiotic Resistance on the Clinical Eradication Results

**Figure 3:**
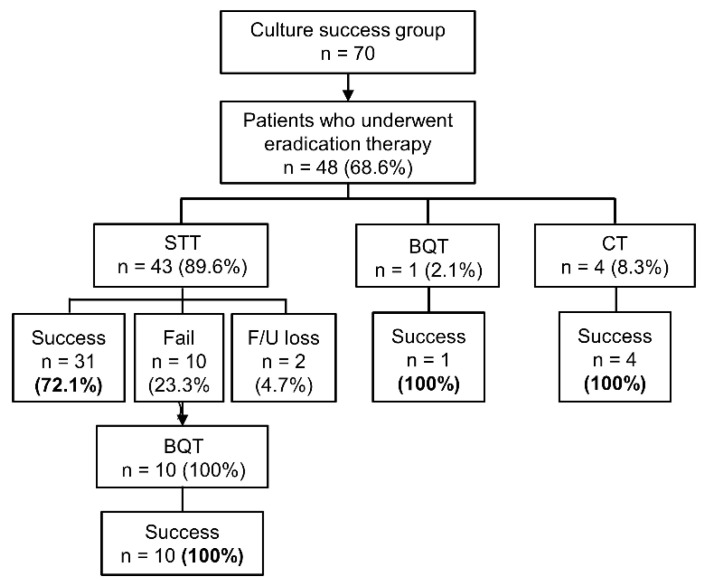
Eradication results of the patients in the culture success group (n = 70). STT, standard triple therapy; BQT, bismuth quadruple therapy; CT, concomitant therapy (proton pump inhibitor + amoxicillin + clarithromycin + metronidazole).

Among 70 patients in the culture success group, 68.6% (48/70) underwent *H. pylori* eradication therapy. Eradication with a first-line treatment was successful in 75% (36/48), while 20.8% (10/48) failed to achieve eradication. Two patients were lost to follow-up. The first-line regimen was STT in 89.6% (43/48), and the second-line treatment was all BQT. Those 10 patients who had failed in the first attempt all achieved successful eradication (10/10) with second-line BQT (Figure 3).

Among 48 patients who underwent eradication therapy, 25% (12/48) were resistant, while 75% (36/48) were sensitive to clarithromycin. The eradication success rate of STT was only 16.7% (2/12) in the clarithromycin-resistant group, which was significantly lower than 93.5% (29/31) in the clarithromycin-sensitive group (*p* < 0.001). Of the 48 patients undergoing eradication therapy, 18.8% (9/48) showed amoxicillin resistance, and 81.3% (39/48) did not. The eradication success rate of STT was 55.6% (5/9) in the amoxicillin-resistant group, whereas it was 76.5% (26/34) in the amoxicillin-sensitive group, and there was no significant difference (*p* = 0.237). Five patients who did not receive STT as the first-line therapy all had strains susceptible to both amoxicillin and clarithromycin. Of them, one underwent BQT and four underwent concomitant therapy, and all of them achieved successful eradication. There were four clarithromycin-sensitive patients with amoxicillin resistance, all of whom had successful eradication with STT.

## 3. Discussion

We investigated the prevalence and the antibiotic resistance pattern of *H. pylori* in Seoul during the last three years (2017–2019). The prevalence of *H. pylori* infection was about 50%. Resistance to major antibiotic agents commonly used for eradication therapy was analyzed. The multidrug resistance, showing resistance to two or more of the target antibiotics, was confirmed in about one-fourth of the subjects. Dual resistance to clarithromycin and metronidazole was confirmed in 8.6% of study subjects with *H. pylori* infection.

The prevalence of *H. pylori* infection is steadily decreasing in Korea. This trend is especially evident among the young population under 40 years of age [13]. Nevertheless, about half the population is still estimated to be infected with *H. pylori* in Korea [14]. The prevalence of *H. pylori* infection was 51.7% in our study, which is in accordance with the data from a recent epidemiologic study [14]. Increasing antibiotic resistance is a major factor that hinders successful *H. pylori* eradication [15]. The Maastricht V guidelines introduced a treatment strategy based on the resistance to clarithromycin and metronidazole, which are the main antibiotics comprising popular eradication regimens [5]. Fluoroquinolone-based regimens can be a reasonable option for rescue therapy, and fluoroquinolone resistance is an important factor in determining the efficacy of these regimens. In order to achieve a high eradication rate of *H. pylori* in real clinical practice, it is crucial to know the current status of resistance to major antibiotics in the community, not only in a timely manner but also accurately.

The clarithromycin resistance was greater than 15%, which is consistent with the results from recent studies conducted in Korea [16,17]. This high resistance is probably related to frequent use of clarithromycin for various indications, including upper or lower respiratory tract infections. In areas with high clarithromycin resistance, STT shows suboptimal eradication results, which is the case in Korea [8,18]. The eradication rate of this regimen has been continuously decreasing, and is now lower than 70% in Korea [19]. Especially, *H. pylori* strains with clarithromycin resistance also showed resistance to amoxicillin in 35% in our study. In these cases with dual resistance, the eradication rate with STT is expected to be very low [20]. Considering that several guidelines oppose the use of STT in areas with high clarithromycin resistance, it is necessary to change the first-line therapy in Korea.

When clarithromycin resistance is anticipated, BQT and non-BQT are both recommended as major alternative treatment options. The resistance to metronidazole is an important factor affecting the efficacy of these regimens [5]. The primary resistance to metronidazole was 25.7% in our study, which is not high compared to other countries [21]. This figure is similar to the one recently reported by Lee et al., which makes our data more reliable [16]. Previous studies from Korea used to report much higher resistance rates to metronidazole before the 2000s, ranging from 33.3 to 66.2% [22,23]. Since the early 2000s, however, this high metronidazole resistance has been gradually decreasing, ranging from 21 to 31.9% [24,25]. The differences in metronidazole resistance might be partially affected by differences in study designs, such as inclusion of patients with previous eradication history, or regional differences of antibiotic resistance. However, there seems to be a clear trend of decreasing resistance to metronidazole in Korea.

Dual resistance to clarithromycin and metronidazole is a major clinical problem, as this can undermine the efficacy of sequential, hybrid, and concomitant therapies, which are representative clarithromycin-based quadruple therapies [26]. Fortunately, this dual resistance was as low as 8.6% in this study, and this value was similar to the calculated value of 7.4% when each resistance was assumed to be independent of each other. This result is highly concordant with that from a recent nationwide mapping study in Korea, which showed a dual resistance rate of 7.2% [16]. Concomitant therapy and BQT can be effective alternatives in regions with high clarithromycin resistance but low dual resistance [5]. A recent randomized controlled study in Korea has shown that the efficacy of 10-day concomitant therapy was 81.2%, which was significantly higher than that of 7-day STT by the intention-to-treat analysis [19]. Recent studies from several European countries also have shown favorable eradication results with concomitant therapy, where the clarithromycin resistance is high while the metronidazole resistance is low to intermediate [27,28].

Generally, it has been widely accepted that the effect of tetracycline resistance on BQT is limited. This was mostly due to the low prevalence of tetracycline-resistant *H. pylori* strains. Interestingly, the resistance to tetracycline was relatively high (18.6%), compared to other previous studies which reported very low resistance rates, ranging from 0 to 5.3% [23,24]. This might be partially explained by the differences in the breakpoint for defining tetracycline resistance. In the past, many researchers used ≥4 mg/mL as a breakpoint, as there were no accredited standards for it. After the European Committee on Antimicrobial Susceptibility Testing (EUCAST) proposed clinical breakpoints of antibiotics for *H. pylori* in 2012, including tetracycline, we can now use the breakpoint of >1 mg/mL, just as in this study [29]. Accordingly, recent studies have also been reporting the increasing trend of tetracycline resistance of *H. pylori* with the same standards for breakpoints [17,30]. The tetracycline resistance more than doubled over 10 years in Korea, by the clinical breakpoint from the EUSCAST [31]. Since tetracycline is a major component of BQT, along with metronidazole, tetracycline resistance can undermine the efficacy of this regimen. To clearly investigate the changing trends of tetracycline resistance of *H. pylori*, it is mandatory to use the unified, reliable standards of breakpoints for resistance when possible.

Multidrug resistance of *H. pylori* was present in more than 40% of the patients. Factors associated with the multidrug resistance include cumulative antibiotic usage, previous treatment failures, and bacterial factors such as mutations, efflux pumps [32]. Since most triple therapies generally comprise a proton pump inhibitor and two different antibiotics, synchronous resistance to two or more antibiotic agents could be a serious clinical problem, by undermining the efficacy of these regimens. This result suggests the importance of performing susceptibility tests to find the adequate combination of antibiotic agents for *H. pylori* eradication.

The clinical data showed that the most common first-line eradication regimen was STT, and the second-line eradication regimen was BQT in every case. The efficacy of STT was lower than 75%, which is definitely suboptimal, as with previous studies. Especially, the efficacy of STT was highly dependent upon the clarithromycin resistance of *H. pylori*, while the amoxicillin resistance did not affect the eradication result significantly. BQT has proven itself to be an acceptable and reliable second-line treatment choice after the failure of first-line STT still in Korea.

There were some limitations in this study. First, this study was conducted in a single tertiary university hospital in Seoul. As regional differences may exist, there could be some differences between the data of this study and the antibiotic resistance of other regions of Korea. Therefore, caution is needed when interpreting and applying the results from this study to the general Korean population. Second, temporal differences in the antibiotic resistance were not analyzed. Nevertheless, providing the information of the up-to-date antibiotic resistance profile of *H. pylori* would be undoubtedly meaningful for the clinical judgment of choosing appropriate eradication regimens.

## 4. Materials and Methods 

### 4.1. Study Population

Subjects older than 19 years old, who required upper endoscopic examination with *H. pylori* test, were prospectively enrolled from May 2017 to December 2019 at Chung-Ang University Hospital, which is a tertiary referral center. The patients with positive *H. pylori* test results were included. The *H. pylori* status was evaluated using the rapid urease test (CLO^®^ test; Kimberly-Clark, UT, USA) or histologic examination at the index upper endoscopy. *H. pylori* infection was considered to be positive if either of these two test results was positive. Patients were excluded if they showed negative results in the *H. pylori* tests, had taken acid-suppressing drugs within two weeks or antibiotics within four weeks, or had a prior history of *H. pylori* eradication. Informed consent was obtained from all participants. The Institutional Review Board of Chung-Ang University Hospital approved this study (approval number: 1610-008-259). The research was conducted in accordance with the ethical standards of the Declaration of Helsinki.

### 4.2. H. pylori Culture and Isolation

During the endoscopic examination, two biopsy samples were taken from the antrum and the body of the stomach, from each patient. The *H. pylori* isolates were cultured at 37 °C for four days under microaerobic conditions (5% O_2_, 10% CO_2_, and 85% N_2_) on Brucella agar plates (Becton Dickinson, Franklin Lakes, NJ, USA) supplemented with 5% defibrinated sheep blood (Hanil Komed, Seongnam, Republic of Korea) [33]. Identification of *H. pylori* was achieved by confirming typical colony morphology, positive rapid urease test, growth on *H. pylori*-selective media [Oxoid™ SR 147 supplement (Thermo Fisher Scientific, Waltham, MA, USA) and 5% defibrinated sheep blood], and detection of urea on PCR. Stock cultures were kept at −70 °C in Brucella broth containing 10% fetal bovine serum and 15% glycerol. For further experiments, these preparations were thawed and subcultured later.

### 4.3. Determination of MICs of Antibiotics

The MIC values of the *H. pylori* isolates to clarithromycin, metronidazole, amoxicillin, tetracycline, levofloxacin, and moxifloxacin were examined using the serial twofold agar dilution method as previously described [33]. In brief, bacteria were subcultured for 48 h on Mueller–Hinton agar plate (Becton Dickinson, NJ, USA) containing 5% defibrinated sheep blood. The bacterial suspension was adjusted to 6 × 10^8^ colony-forming units/mL (McFarland Standard No.2), and subsequently inoculated onto the agar dilution plates containing the dilutions of each antibiotic agent. Following incubation for 72 h, the MIC of each antibiotic agent was determined. *H. pylori* ATCC 43504 was used for quality control. The resistance breakpoints for these six antibiotics were defined as >0.5, >8.0, >0.125, >1.0, >1.0, and >1.0 μg/mL, respectively, in accordance with the EUCAST standards [29]. The above procedures were performed in triplicate and repeated three times to determine the MICs of target antibiotics. The resistance rate to each antibiotic was calculated by dividing the number of patients with the strain showing resistance by the total number of patients with successful culture result. The multidrug resistance was defined as showing resistance to two or more classes of antimicrobial agents. Resistance to either or both of levofloxacin and moxifloxacin was considered as single resistance to fluoroquinolone class, when calculating the multidrug resistance.

### 4.4. Data Collection of Eradication Results

Clinical data of the patients, such as eradication regimen, success rate, and total number of eradication attempts, were investigated by reviewing the electronic medical records. Follow-up test for the confirmation of eradication comprised urea breath test (UBiT-IR 300^®^, Otsuka Pharmaceutical Co. Ltd., Tokyo, Japan), rapid urease test (CLO^®^ test, Kimberly-Clark, UT, USA), or histologic evaluation with Wright–Giemsa staining. One or more of these tests were performed at the physician’s discretion, at least four weeks after the completion of the eradication treatment. A positive finding of any of the follow-up tests was regarded as eradication failure, and successful eradication was defined as showing negative results from all follow-up tests performed.

### 4.5. Statistical Analysis

Categorical variables were compared using the Fisher’s exact test. *p* value < 0.05 was considered statistically significant. All statistical analyses were performed using SPSS version 19 (SPSS Inc., Chicago, IL, USA).

## 5. Conclusions

The primary resistance rates of *H. pylori* to clarithromycin were high, yet the prevalence of dual resistance to clarithromycin and metronidazole was still low in Korea. STT is no longer an acceptable first-line option for *H. pylori* eradication in Korea. The increasing rate of multidrug resistance implies the importance of antibiotic susceptibility tests and the need for a cautious approach in the selection of appropriate eradication regimens.

## Figures and Tables

**Table 1:** Baseline characteristics of the study population.

Variables	Total Enrolled Patients	Patients with Successful *H. pylori* Culture (n = 70)
	(n = 174)	
Number of patients	174 (100%)	70 (100%)
Mean age ± SD (years)	60.5 ± 13.1	61.2 ± 9.2
Sex (male/female)	100/74 (57.5%/42.5%)	44/36 (62.9%/37.1%)
Endoscopic diagnosis		
Peptic ulcer disease	34 (19.5%)	20 (28.6%)
Gastric cancer	34 (19.5%)	13 (18.6%)
Gastric adenoma	17 (9.8%)	5 (7.1%)
MALT lymphoma	1 (0.6%)	1 (1.4%)
Gastritis	88 (50.6%)	31 (44.3%)

Values are shown as number (%) or mean ± SD unless stated otherwise. SD, standard deviation; MALT, mucosa-associated lymphoid tissue.

**Table 2:** Multidrug resistance profile of *Helicobacter pylori.*

Drug Resistance Profile					Subjects with Successful *H. pylori* Culture (n = 70)	Subjects with Resistant *H. pylori* Strains (n = 49)
Resistant to 5 drugs					1 (1.4%)	1 (2.0%)
**C**	M	A	T	Q	1 (1.4%)	1 (2.0%)
Resistant to 4 drugs					2 (2.9%)	2 (4.1%)
C	M	A		Q	1 (1.4%)	1 (2.0%)
	M	A	T	Q	1 (1.4%)	1 (2.0%)
Resistant to 3 drugs					10 (14.3%)	10 (20.4%)
C	M		T		1 (1.4%)	1 (2.0%)
C		A	T		1 (1.4%)	1 (2.0%)
C		A		Q	4 (5.7%)	4 (8.2%)
	M	A		Q	2 (2.9%)	2 (4.1%)
	M		T	Q	2 (2.9%)	2 (4.1%)
Resistant to 2 drugs					17 (24.3%)	17 (34.7%)
C	M				3 (4.3%)	3 (6.1%)
C				Q	4 (5.7%)	4 (8.2%)
	M			Q	4 (5.7%)	4 (8.2%)
		A		Q	2 (2.9%)	2 (4.1%)
			T	Q	4 (5.7%)	4 (8.2%)
Resistant to 1 drug					19 (27.1%)	19 (38.8%)
C					5 (7.1%)	5 (10.2%)
	M				4 (5.7%)	4 (8.2%)
		A			2 (2.9%)	2 (4.1%)
			T		3 (4.3%)	3 (6.1%)
				Q	5 (7.1%)	5 (10.2%)

Values are shown as number (%). C, clarithromycin; M, metronidazole; A, amoxicillin; T, tetracycline; Q, fluoroquinolone.

**Table 3:** Multidrug resistance profile of *Helicobacter pylori*, excluding fluoroquinolones.

Drug Resistance Profile				Subjects with Successful *H. pylori* Culture (n = 70)	Subjects with Resistant *H. pylori* Strains (n = 44)
Resistant to 4 drugs				1 (1.4%)	1 (2.3%)
C	M	A	T	1 (1.4%)	1 (2.3%)
Resistant to 3 drugs				4 (5.7%)	4 (9.1%)
C	M	A		1 (1.4%)	1 (2.3%)
C	M		T	1 (1.4%)	1 (2.3%)
C		A	T	1 (1.4%)	1 (2.3%)
	M	A	T	1 (1.4%)	1 (2.3%)
Resistant to 2 drugs				11 (15.7%)	11 (25.0%)
C	M			3 (4.3%)	3 (6.8%)
C		A		4 (5.7%)	4 (9.1%)
	M	A		2 (2.9%)	2 (4.5%)
	M		T	2 (2.9%)	2 (4.5%)
Resistant to 1 drug				28 (40.0%)	28 (63.6%)
C				9 (12.9%)	9 (20.5%)
	M			8 (11.4%)	8 (18.2%)
		A		4 (5.7%)	4 (9.1%)
			T	7 (10.0%)	7 (15.9%)

Values are shown as number (%). C, clarithromycin; M, metronidazole; A, amoxicillin; T, tetracycline.

## Supplementary Materials

The following are available online at https://www.mdpi.com/2079-6382/9/10/646/s1, Table S1: Differences in the resistance of *Helicobacter pylori* strains from the antrum and the body for each antibiotic agent (*n* = 52), Table S2: MIC levels and resistance profiles of *Helicobacter pylori* strains of the included patients (*n* = 70)
