# Supplementary material for: The Prevalence of Multidrug Resistance of Helicobacter pylori and Its Impact on Eradication in Korea from 2017 to 2019: A Single-Center Study
Source: Antibiotics (Basel). 2020 Sep 27;9(10):646. doi: 10.3390/antibiotics9100646 (PMC7601770; doi:10.3390/antibiotics9100646)
Supplement: Supplementary file 1 [file antibiotics-09-00646-s001.zip › Supplementary materials_Table S1.pdf]

antibiotics

Table S1 Differences in the resistance of *Helicobacter pylori* strains from the antrum and the body for each antibiotic agent (n=52).

Antibiotic agents	Resistance (Antrum)	Resistance (Body)	Discordant resistance pattern*
Clarithromycin	17 (32.7%)	17 (32.7%)	2 (3.8%)
Metronidazole	12 (23.1%)	15 (28.8%)	3 (5.8%)
Amoxicillin	12 (23.1%)	12 (23.1%)	0 (0%)
Tetracycline	5 (9.6%)	9 (17.3)	8 (15.4%)
Levofloxacin	24 (46.2%)	23 (44.2%)	3 (5.8%)
Moxifloxacin	24 (46.2%)	23 (44.2%)	3 (5.8%)

Values are shown as number (%).

*Discordant resistance pattern means that there is a difference in the presence of antibiotic resistance between the strains from the antrum and the body in a single host.
